# Soluble iron modulates iron oxide particle-induced inflammatory responses via prostaglandin E_2 _synthesis: *In vitro *and *in vivo *studies

**DOI:** 10.1186/1743-8977-6-34

**Published:** 2009-12-22

**Authors:** Ingrid Beck-Speier, Wolfgang G Kreyling, Konrad L Maier, Niru Dayal, Mette C Schladweiler, Paula Mayer, Manuela Semmler-Behnke, Urmila P Kodavanti

**Affiliations:** 1 Comprehensive Pneumology Center, Institute of Lung Biology and Disease, German Research Center for Environmental Health, D-85764 Neuherberg, Germany; 2 Focus Network Nanoparticles and Health, Helmholtz Center Munich - German Research Center for Environmental Health, D-85764 Neuherberg, Germany; 3 Environmental Public Health Division, National Health and Environmental Effects Research Laboratory, US Environmental Protection Agency, Research Triangle Park, NC 27711, USA

## Abstract

**Background:**

Ambient particulate matter (PM)-associated metals have been shown to play an important role in cardiopulmonary health outcomes. To study the modulation of PM-induced inflammation by leached off metals, we investigated intracellular solubility of radio-labeled iron oxide (^59^Fe_2_O_3_) particles of 0.5 and 1.5 μm geometric mean diameter. Fe_2_O_3 _particles were examined for the induction of the release of interleukin 6 (IL-6) as pro-inflammatory and prostaglandin E_2 _(PGE_2_) as anti-inflammatory markers in cultured alveolar macrophages (AM) from Wistar Kyoto (WKY) rats. In addition, we exposed male WKY rats to monodispersed Fe_2_O_3 _particles by intratracheal instillation (1.3 or 4.0 mg/kg body weight) to examine *in vivo *inflammation.

**Results:**

Particles of both sizes are insoluble extracellularly in the media but moderately soluble in AM with an intracellular dissolution rate of 0.0037 ± 0.0014 d^-1 ^for 0.5 μm and 0.0016 ± 0.0012 d^-1 ^for 1.5 μm ^59^Fe_2_O_3 _particles. AM exposed *in vitro *to 1.5 μm particles (10 μg/mL) for 24 h increased IL-6 release (1.8-fold; p < 0.05) and also PGE_2 _synthesis (1.9-fold; p < 0.01). By contrast, 0.5 μm particles did not enhance IL-6 release but strongly increased PGE_2 _synthesis (2.5-fold, p < 0.005). Inhibition of PGE_2 _synthesis by indomethacin caused a pro-inflammatory phenotype as noted by increased IL-6 release from AM exposed to 0.5 μm particles (up to 3-fold; p < 0.005). In the rat lungs, 1.5 but not 0.5 μm particles (4.0 mg/kg) induced neutrophil influx and increased vascular permeability.

**Conclusions:**

Fe_2_O_3 _particle-induced neutrophilic inflammatory response *in vivo *and pro-inflammatory cytokine release *in vitro *might be modulated by intracellular soluble iron via PGE_2 _synthesis. The suppressive effect of intracellular released soluble iron on particle-induced inflammation has implications on how ambient PM-associated but soluble metals influence pulmonary toxicity of ambient PM.

## Background

Ambient respirable particles vary in their size, chemical composition, and surface characteristics. The toxicity of PM differs based on the bioavailability of components loosely adherent to particles and the surface chemistry [1]. Because ambient particles are heterogeneous due to their diverse origin, the study of the contribution of each component in causing lung cell response has been challenging. Particulate matter (PM) exposure causes pulmonary inflammation and also cardiovascular health impact [2,3]. Often a homogenous laboratory made respirable particle preparation is used to delineate the role of each component/characteristics in eliciting pulmonary and cardiovascular effects. While it is postulated that the type of initial pulmonary injury caused by PM influences cardiovascular effects, it is necessary to study pulmonary inflammatory potential of leached-off and adherent PM components.

In this context, alveolar macrophages (AM) representing the first line of pulmonary defense might play a central role. As part of the innate immune system, they eliminate invading pathogens and particles by phagocytosis, produce reactive oxygen species and thereby release cytokines and lipid mediators to manage inflammatory processes [4-6]. Among the lipid mediators, prostaglandin E_2 _(PGE_2_) predominantly exhibits immune-modulating functions that limit inflammatory responses and control tissue repair [7]. Ultrafine particles of different sizes are able to activate the synthesis of lipid mediators such as PGE_2 _[8,9]. Physicochemical properties are likely to impact the mechanism by which pulmonary lipid mediators and pro-inflammatory cytokine-mediated inflammation occurs following exposure to PM.

Water-leachable particulate matter (PM)-associated transition metals have been shown to be one of the causative components involved in acute pulmonary and cardiovascular health effects [10-12]. Transition metals are frequent contaminations of PM (such as residual oil fly ash) that can mediate direct toxic effects on pulmonary epithelium and macrophages [13,14]. However, the mechanism by which each leached-off metal may modulate particle-core effects on alveolar macrophages and ultimate inflammatory responses are not clearly understood. In addition to having direct cellular effects these loosely-bound or leachable metals may influence the macrophage phagocytosis of insoluble particle core and the ultimate inflammatory responses.

Iron is the ubiquitous transition metal found in greatest abundance in ambient PM [15]. At high concentration in different chemical forms and in the presence of reducing equivalents in lung lining fluid, iron can initiate Fenton-like reactions and generate highly reactive oxygen-derived free radicals [16]. This oxidative stress resulting from the interaction of the lung epithelium with catalytically-active iron on the surface of ambient PM could promote adverse health effects. However, such high concentrations of soluble or surface reactive iron are less likely to be achieved by inhalation of ambient particles. To prevent toxic effects by catalytically-active cellular iron, lungs possess the ability to transport and sequester free (as ferric [Fe^3+^] form) iron in an inactive form using iron binding proteins [17]. However, it is not known how relatively small amounts of leached-off iron within phagocytes may bind proteins and initiate cell signaling which ultimately influences particle core-induced inflammatory responses in the lung. To study the mechanisms by which particle-associated soluble iron modulate inflammatory responses induced by solid particle core, we used physicochemical uniform iron oxide (Fe_2_O_3_) particles of two different sizes.

Fe_2_O_3 _particles have low solubility in aqueous media but can be more soluble in acidic milieu, such as within alveolar macrophages (AM) [18]. We have shown earlier that intracellular solubility depends on the specific surface area of the particles such that small particles with large specific surface areas are more rapidly dissolved than larger particles with smaller specific surface areas [18,19]. Thus, the use of such particle samples of different size provides the opportunity to investigate how core particle-induced inflammatory response might be modulated by the amount of metal that is leached. Recently nano-sized iron oxide particles have been shown to induce lung inflammation at high intratracheal instillation doses and have been shown to translocate systemically, with a major portion remaining in the lung several months post exposure [20,21]. In the present study, we hypothesized that soluble iron within AM plays a role in modulating Fe_2_O_3 _particle core-induced inflammatory responses *in vitro *and *in vivo*. We further postulated that the level of prostaglandin E_2 _(PGE_2_) induction will modulate inflammation by its anti-inflammatory action. Low cytotoxicity of carbonyl iron particles has been speculated to be due to iron modulation of macrophage PGE_2 _production [22].

We selected physicochemical uniform Fe_2_O_3 _particles of two different sizes (1.5 μm and 0.5 μm) and corresponding surface areas (7.1 m^2^/g and 17 m^2^/g) for their extra- and intracellular solubility and the ability to activate inflammatory reactions *in vitro *and *in vivo*. We believed that phagocytosed small Fe_2_O_3 _particles would yield more soluble iron than large particles when incubated with AM at an equivalent mass basis. Particles of these sizes (with sufficient solubility differences) can be effectively phagocytosed by macrophages to allow the study of the role of leached iron in modulating the iron core particle's inflammatory responses. Particle dissolution rates and production of IL-6 (a pro-inflammatory cytokine) and PGE_2 _(an anti-inflammatory mediator) were examined using rat AM. The inflammatory effect of the Fe_2_O_3 _particles was also studied *in vivo *after intratracheal instillation into the lungs of healthy Wistar Kyoto (WKY) rats. Our data demonstrate that the amount of soluble iron released intracellularly from phagocytosed Fe_2_O_3 _particles might modulate PGE_2 _production and pro-inflammatory cytokine release *in vitro*, and inflammation in the lung *in vivo*.

## Materials and methods

### Materials

Phosphate buffered saline (PBS) with and without Ca^2+^/Mg^2+ ^was purchased from Biochrome (Berlin, Germany); RPMI was from PAA Laboratories (Linz, Austria); fetal calf serum, penicillin, streptomycin and amphotericin were from Life Technologies (Eggenstein, Germany); all other chemicals (analytical or HPLC grade were from Merck (Darmstadt, Germany).

### Animals

Twelve- to -14-wk-old, male Wistar Kyoto rats (WKY/Kyo@Rj; Janvier, France) were used for bronchoalveolar lavage to provide alveolar macrophages (AM) for the *in-vitro *studies. These rats were housed in pairs in a humidity- (55% relative humidity) and temperature- (22°C) controlled room in individually ventilated cages (Ventirack™, cage type CU-31), maintained on a 12-h day/night cycle prior to the study. Laboratory animal diet and water was provided *ad libitum*. The studies were conducted under Federal guidelines for the use and care of laboratory animals and were approved by the Regierung von Oberbayern (District of Upper Bavaria, Approval No. 211-2531-108/99 for rats) and by the GSF Institutional Animal Care and Use Committee. Twelve- to -14-wk-old, male WKY rats were also used for *in vivo *intratracheal instillation studies (Charles River, Raleigh, NC, USA). These animals were housed in an isolated animal room in the AAALAC-approved animal facility (21 ± 1°C, 50 ± 5% relative humidity, 12 h light-dark cycle) and allowed free access to standard 5001 Purina Rat Chow (Brentwood, MO) and water. Institutional (EPA) Animal Care and Use Committee approved the study protocol prior to the start of the study.

### Production of Fe_2_O_3 _particles

Two batches of monodisperse ferric oxide (Fe_2_O_3_) particles were produced from a solution of ferric nitrate (Fe_2_(NO_3_)_3_) dispersed into droplets of the appropriate concentration and uniform size by using a spinning top aerosol generator. Droplets were dried at 140°C prior to on-line thermal degradation at 800°C in a tube furnace. Particles were prepared with and without labeling by ^59^Fe. During generation, the aerodynamic characterization of the particles was continuously monitored with an aerodynamic particle sizer (Type APS33, TSI Inc.) and by low-angle forward-scattering optical aerosol spectrometry [23]. The physical size (count median diameter, CMD) of the particles was determined by scanning electron microscopy (SEM). The particles were spherical, with rough surface area as also confirmed by transmission electron microscopy (TEM). Density was calculated from the median aerodynamic diameter and CMD. Specific surface area was determined from TEM images and from BET measurements of physicochemical uniform cobalt oxide (Co_3_O_4_), terbium oxide (Tb_4_O_7_) and gadolinium oxide (Gd_2_O_3_) particles of similar size and produced earlier under same conditions [18,24-27].

### Alveolar macrophages (AM) for in vitro study

AM from healthy WKY rats were isolated by bronchoalveolar lavages (repeated 5 times) using fresh aliquots of Ca^2+^/Mg^2+^-free PBS kept at 37°C each time at a volume equivalent to 28 mL/kg total lung capacity. After 20 min centrifugation at 400 *g *cells were resuspended in RPMI medium containing penicillin (100 U/mL), streptomycin (100 U/mL), amphotericin (2.5 μg/mL) and 5% fetal calf serum. Viability of cells was about 95% as estimated by trypan blue exclusion. After May Grünwald Giemsa staining of cytospin preparations microscopic examination identified about 95-99% of cells as AM. The RPMI medium containing penicillin (100 U/mL), streptomycin (100 U/mL), amphotericin (2.5 μg/mL) and 5% fetal calf serum, referred as full medium below, was used for all the *in vitro *incubations of cells with particles and control cells without particles.

### Determination of intracellular particle dissolution (IPD) by alveolar macrophages (AM)

Rat AM (1 × 10^5 ^cells/well) were incubated with ^59^Fe-labeled 0.5 and 1.5 μm Fe_2_O_3 _particles in 96 well plates for 12 days at 37°C, and the IPD of ^59^Fe_2_O_3 _particles in AM was determined according to Kreyling *et al *[19]. Briefly, AM were incubated in full medium using a monolayer technique in 96 well plates. AM were purified by media exchange 2-4 hours later. With the new media ^59^Fe_2_O_3 _particles were added at a particle to AM ratio of 1:1. AM covered 5-10% (10^5 ^per well) of the well bottom. These conditions allowed maintenance of fully functional AM through the time of incubation without exchange of media as had been optimized before. As described earlier in detail [19] complete phagocytosis of the ^59^Fe_2_O_3 _particles (non-visible at the highest magnification ×600 of the inverted microscope) within 24 hours was monitored by quantifying phagocytosis of 2 μm fluorescent latex particles (FPSL) using an inverted microscope. For this test FPSL were added to two separate wells at a FPSL to AM ratio of 1:1. Complete phagocytosis was assumed after 24 hours when more than 90% of the FPSL were associated with AM as oppose to only 10% after 30 min. The functional state of the AM was monitored during seven days of incubation by determining the cell concentration and viability in separate wells. IPD measurements were excluded when any of the *in vitro *functions of AM were markedly impaired.

During the next 7 days at 4 time points, intracellular dissolved ^59^Fe was determined by gamma spectroscopy in filtrates containing the dissolved ^59^Fe versus particulate ^59^Fe_2_O_3 _on filters of both the medium and the cell lysate. First the cell culture medium was filtered (0.22 μm membrane filter, Millipore, Schwalbach, Germany) providing the dissolved ^59^Fe in the filtrate AD_med _(t) and the particulate ^59^Fe_2_O_3 _AP_med _(t); then the cells were lysed in the wells and subsequently the suspension of cell debris was filtered, too. The filtrates of the cell lysates represented intracellular dissolved iron AD_lys _(t) which was retained intracellularly while the filters contained the rest of particulate ^59^Fe_2_O_3 _(AP_lys _(t). Dissolved and particulate iron fractions DF_j _and PF_j _were obtained by normalizing activities AD_j _and AP_j _by the sum of all four samples at a time t:(1)

**Figure 1:**
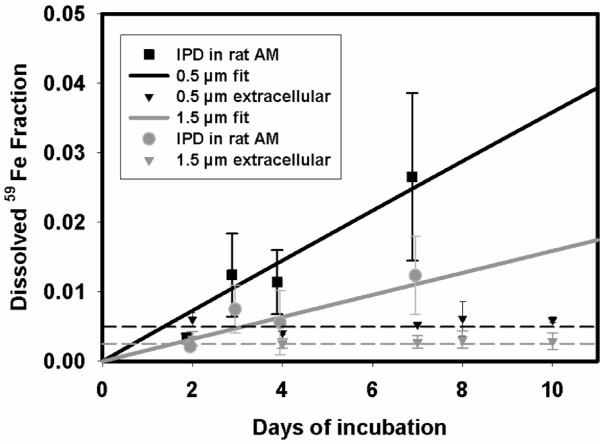
**Intracellular and extracellular dissolution of 1.5 and 0.5 μm ^59^Fe_2_O_3 _particles in alveolar macrophages (*in vitro *study)**. Rat alveolar macrophages (AM) or incubation media without AM were incubated in vitro with ^59^Fe-labeled 0.5 μm Fe_2_O_3 _particles and 1.5 μm Fe_2_O_3 _particles, respectively. The intracellular particle dissolution (IPD) rate for cells of 4 different animals (n = 4) and the extracellular dissolution for 4 different incubations without cells (n = 4) were determined over a period of 7 days. The small 0.5 μm particles exhibited a higher IPD rate (0.0037 ± 0.0014 d-1; n = 4) than the large 1.5 μm particles (0.0016 ± 0.0012 d-1; n = 4); both rates were corrected for extracellular particle dissolution see equation 3. The extracellular dissolution rate for both types of particles was negligible small.

From the measured dissolution kinetics of time-dependently increasing dissolved ^59^Fe fractions of the sum of AM medium and lysate, a mean total IPD rate and standard error were calculated, see Figure 1. For control, extracellular dissolved ^59^Fe concentrations DF_ext _(t) were determined in parallel in full media without cells by filtrations at the same time points t in order to determine the dissolved fractions DF_ext _(t) of extracellular particulate fractions PF_ext _(t). From the measured dissolution kinetics of time-dependently increasing dissolved fractions of the sum of AM medium and lysate, a mean total IPD rate and standard error were calculated, see Figure 1.(3)

Note that dissolved factions DF_med _(t_i_) + DF_lys _(t_i_) were already corrected for externally dissolved DF_ext _(t_i_). Data and the fitted function of both particle sizes are shown in Figure 1. The control data of extracellular ^59^Fe dissolution in full media DF_ext _(t) showed no time dependency but a small immediate leaching effect at the beginning of incubation.

In order to determine the uptake of free extracellular iron by cultured AM, additional incubations were done and the cellular uptake of Fe^2+ ^from medium into AM was measured. AM were incubated in 96 well-plates at 10^5 ^per well (0.2 ml) and iron chloride (FeCl_2_) radio-labeled with ^59^Fe was added varying between 0.1 - 5.0 μg FeCl_2_/ml or 10^6 ^AM.

### Analysis of IL-6 release and intracellular PGE_2 _synthesis

For the simultaneous determination of intracellular PGE_2 _synthesis and IL-6 release, AM of WKY rats (0.5 × 10^6 ^cells/0.5 mL) were incubated with or without 10 μg/mL of either 0.5 or 1.5 μm Fe_2_O_3 _particles in 48 well plates for 24 h at 37°C. Control cells were incubated under the same conditions in parallel. After incubation of the cells, the conditioned supernatants were isolated and stored at -80°C for measurement of IL-6 release; the cells were then deproteinized by addition of an eight-fold volume of 90% methanol (containing 0.5 mM EDTA and 1 mM 4-hydroxy-2,2,6,6-tetramethylpiperidine-1-oxide [pH 7.4]) to assess intracellular PGE_2 _content [9] and protein determination. The deproteinized cell suspensions were stored at -40°C for 24 h followed by a two-time centrifugation at 10000 *g *for 20 min at 4°C with a 24 h interval to precipitate the protein. Aliquots of the deproteinized fractions were dried in a vacuum centrifuge, and used for determination of PGE_2_. For protein determination, the precipitated proteins were dried under nitrogen to remove any methanolic solution, resuspended in half of the volume in Hepes, pH 7.4, sonicated (3 times for 15 sec) and centrifuged at 10000 *g*. The supernatants were taken for protein determination, measured with a 1:5 diluted Bio-Rad solution (Bio-Rad, Munich, Germany) at 595 nm using bovine serum albumin as standard. For inhibition experiments with indomethacin, the cells were pretreated with 100 μM indomethacin for 15 min and subsequently incubated with or without particles for 24 h.

The intracellular content of PGE_2 _was determined in the deproteinized supernatants. Aliquots of the deproteinized supernatants were dried in a vacuum centrifuge and used for measuring PGE_2 _by a PGE_2 _specific enzyme immunoassay according to the instructions of the manufacturer (Cayman, Ann Arbor, MI, USA).

The IL-6 release from particle-treated AM in the conditioned supernatants was quantified by a rat specific IL-6 ELISA according to the instructions of the manufacturer (Bender MedSystems, Vienna, Austria).

### Intratracheal instillation of Fe_2_O_3 _particles into the lungs of WKY rats

In order to compare relative inflammatory potential of each size particle, we selected two doses with three-fold difference in the mass concentration. This allowed us to compare similar surface area dose (1.3 mg of 0.5 μm particles and 4.0 mg of 1.5 μm particles) for at least one dose level. Although these particles do not represent the composition of ambient PM in the surface characteristics, we believe that these particles provide controlled experimental condition where the role of solubility of iron can be determined. Although the concentrations we used are orders of magnitude higher that one would encounter environmentally at one time, these are the concentrations that caused moderate degree of pulmonary injury and inflammation without the overt toxicity. The dose levels of 1.5 and 4.0 mg/kg body weights were also selected based on the understanding that synthetic iron oxide particles will cause relatively mild inflammatory response in the lungs [28]). Both 0.5 and 1.5 μm Fe_2_O_3 _particles were suspended in sterile saline at two concentrations: 1.3 mg/mL or 4.0 mg/mL, respectively. The WKY rats were then intratracheally instilled at 1.0 mL/kg under halothane anesthesia [29]. Control rats received 1 mL/kg sterile saline only.

### Analysis of bronchoalveolar lavage fluid (BALF)

Bronchoalveolar lavages of control and exposed rats were done at 24 h after instillation according to Kodavanti *et al*. [13]. Briefly, rats were anesthetized with sodium pentobarbital (Nembutal, Abott Lab., Chicago, USA; 50-100 mg/kg body weight, intraperitoneal) and exsanguinated via abdominal aorta. The tracheas were cannulated. The lungs were lavaged using Ca^2+ ^and Mg^2+^-free phosphate-buffered saline (PBS; pH 7.4) at a volume of 28 mL/kg body weight. Three in-and-out washes were performed using the same fluid. One aliquot of lavage fluid was used for determining total cells using a Coulter Counter (Coulter, Inc., Miami, USA), and a second aliquot was centrifuged using a Shandon 3 Cytospin (Shandon Inc., Pittsburgh, PA, USA) for preparing cell differential slides. After drying at room temperature the slides were stained with LeukoStat (Fisher Scientific Co., Pittsburgh, PA, USA). Macrophages, neutrophils, eosinophils and lymphocytes were quantified using light microscopy (200 cells/slide).

The remaining BALF was centrifuged at 1500 *g *to remove cells, and the supernatant fluid was analyzed for protein, albumin and lactate dehydrogenase (LDH) activity. Assays for each endpoint were modified and adapted for use on a Hoffmann-La Roche Cobas Fara II clinical analyzer (Roche Diagnostics, Indianapolis, IN, USA). Total protein content was determined using a Coomassie Plus Protein Assay Kit (Pierce, Rockford, IL, USA) with bovine serum albumin as standard. The albumin content was analyzed using a commercially available kit (ICN Star Corporation (Stillwater, MN, USA). LDH activity was determined using Kit 228 and standards from Sigma Chemicals Co. (St. Louis, MO, USA).

### Statistical analysis

Statistical significance was determined by analysis of variance and two-sample t-test. Changes with P < 0.05 were considered significant.

## Results

### Characteristics of Fe_2_O_3 _particles

**Figure 2:**
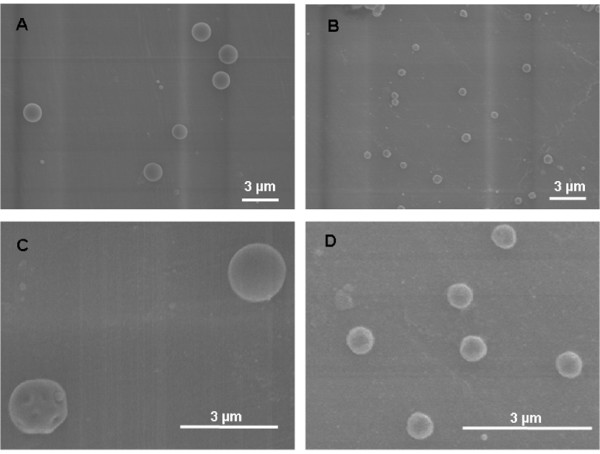
**Scanning electron microscope (SEM) pictures of the 1.5 μm and 0.5 μm Fe_2_O_3 _particles**. SEM of 1.5 μm Fe_2_O_3 _particles (panels A + C) and of 0.5 μm Fe_2_O_3 _particles (panels B + D); magnifications of original SEM pictures in panels A + B are 5K; magnification of close-ups of SEM pictures in panel C is 13K and in panel D is 17K. The larger magnifications show the rough surface area of the spherical Fe_2_O_3 _particles of both sizes as an indicator for their moderate porosity.

The physical characteristic of the two types of monodisperse Fe_2_O_3 _particles are listed in Table 1. The 1.5 μm particles have a surface area of 7 m^2^/g, whereas the smaller 0.5 μm particles have a larger surface area of 17 m^2^/g. Density was calculated from the measured aerodynamic median and the physical median diameter to be 3.8 g/cm^3 ^for both particle sizes. In addition to Table 1, Figure 2 shows SEM images of both types of particles each at two magnifications. The large 1.5 μm particles (Figure 2A) with their magnification (Figure 2C) and the small 0.5 μm particles (Figure 2B) with their magnification (Figure 2D) show very well the spherical and porous structure of the particles and their rough surface area.

**Table 1:** Physical characteristics of Fe_2_O_3 _particles

Physical Characteristics	Large Particles	Small Particles
Geometric median diameter (μm)	1.5	0.5

Aerodynamic median diameter (μm)	2.8	1.3

Geometric Standard Deviation	1.2	1.2

Density (g/cm^3^)	3.8	4.1

Estimated surface area (m^2^/g)	7.1	17

The monodisperse Fe_2_O_3 _particles were produced using a spinning top aerosol generator with subsequent online heat degradation of the particles in a tube furnace at 800°C. The aerodynamic characterization of the particles was continuously monitored with an aerodynamic particle sizer (Type APS33, TSI Inc.) and by low-angle forward-scattering optical aerosol spectrometry.

### Extra- and intracellular particle dissolution rate of 0.5 μm and 1.5 μm Fe_2_O_3 _particles

In a cellular *in vitro *system with rat AM both types of ^59^Fe_2_O_3 _particles were evaluated for their extra- and intracellular dissolution rates over 10 days (Figure 1). In the medium without AM, a small dissolved iron fraction occurred initially, but particles did not dissolve any more thereafter suggesting that the extracellular dissolved fraction remains constant during the incubation time. The extracellular dissolved fraction of the incubated 0.5 μm particles was 0.00055 ± 0.00022 and for 1.5 μm particles was 0.00034 ± 0.00016. In contrast, the intracellular dissolved fractions increased with time. The intracellular particle dissolution (IPD) rates from 0.5 and 1.5 μm ^59^Fe_2_O_3 _particles were 0.0037 ± 0.0014 d^-1 ^and 0.0016 ± 0.0012 d^-1^, respectively. From the least square fitted data these rates differed significantly (p < 0.01) between both particle sizes; in addition, when applying least square fits to the extracellular dissolved fractions of both particle sizes, those rates were very close to zero and differed significantly (p < 0.001) to the IPD rates of the two particle sizes.

From the particle dissolution kinetics data determined from measures of iron in the culture medium and cell lysate (corrected for external dissolved Fe), we found that more than 70% of the dissolved iron remained within AM (likely iron binding protein-associated), whereas only 30% was released out of the cell into the extracellular medium. In fact, we cannot exclude trafficking of dissolved Fe back and forth across AM membranes according to the complex iron metabolism since we only measure the net effect. In comparison, our recent studies on moderately soluble cobalt oxide particles [19] showed that the intracellular dissolved and retained cobalt was only 5-10% since most dissolved cobalt was released rapidly out of the AM. This is in contrast to the fate of the dissolved iron in this study since it was predominantly retained - most likely associated with iron binding proteins - in AM. Furthermore, extracellular iron Fe^2+ ^at doses between 0.1-5 μg/mL medium (or per 10^6 ^AM) was taken up relatively weakly by AM (24 ± 2.5% of any of the doses provided). Hence, these data show that the dissolved iron from phagocytosed particles was primarily retained intracellularly while relatively small fractions of external dissolved iron were sequestered by AM. After one week the intracellular dissolved and retained iron progressively increased to about 3% with small and 1% with large ^59^Fe_2_O_3 _particles (Figure 1). This amount of intracellular available soluble iron is important for the cellular responses as shown below.

### Cellular effects of 1.5 μm and 0.5 μm Fe_2_O_3 _particles: release of IL-6 and synthesis of PGE_2_

**Figure 3:**
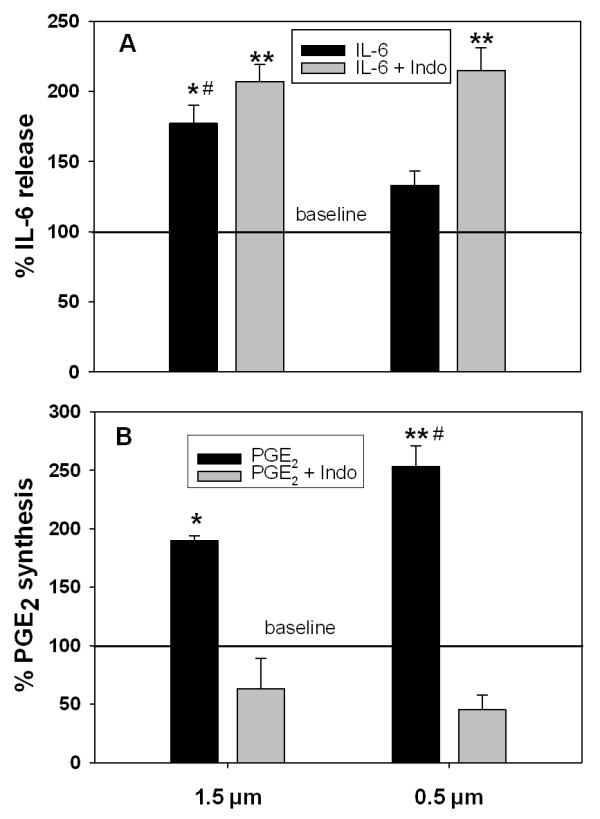
**IL-6 release and PGE_2 _synthesis in alveolar macrophages by 1.5 and 0.5 μm Fe_2_O_3 _particles (*in vitro *study)**. Rat alveolar macrophages were incubated with large 1.5 μm or small 0.5 μm Fe_2_O_3 _particles for 24 h in RPMI, respectively, and studied in absence and presence of PGE_2 _synthesis inhibitor indomethacin: (A) release of IL-6 as pro-inflammatory mediator, and (B) synthesis of PGE_2 _as anti-inflammatory and immune-modulating mediator. Data are given as mean ± SD of the percentages for IL-6 release and PGE_2 _synthesis obtained from cells of 4 different animals (n = 4). Baseline value represents 100% of IL-6 release with 304 ± 105 pg/mL (n = 4), and 100% for PGE_2 _synthesis with 408 ± 140 pg/mL (n = 4). P-values (* P < 0.05; ** P < 0.005) for significant difference between baseline and particle-treated cells in the absence and presence of indomethacin and (# P < 0.01) for significant difference between large 1.5 μm and small 0.5 μm Fe_2_O_3 _particles in absence of indomethacin for IL-6 release (A) and PGE_2 _synthesis (B).

Both types of Fe_2_O_3 _particles were evaluated for their capacity to produce and release inflammatory and anti-inflammatory mediators such as IL-6 and PGE_2 _from rat AM. To elucidate whether one of these parameters affect the other, the AM incubations with particles were performed in the absence and presence of indomethacin, an inhibitor of PGE_2 _synthesis. IL-6 release in the media and PGE_2 _in cells were analyzed with or without one of the two sized iron oxide particles. As shown in Figure 3A, in the absence of indomethacin the release of IL-6 was significantly induced by AM exposed to the large 1.5 μm particles (1.8-fold; *P < 0.05) but not by those treated with the small 0.5 μm particles. In the presence of indomethacin the IL-6 release increased strongly by both types of particles (**P < 0.005) compared to non-particle baseline. The significant difference in IL-6 release between the large and the small particles in the absence of indomethacin (#P < 0.01) was not observed in the presence of indomethacin. Figure 3B shows that in the absence of indomethacin the intracellular PGE_2 _synthesis is enhanced by the large (1.9-fold; *P < 0.05) and even more by the small particles (2.5-fold; **P < 0.005) compared to non-particle baseline. Moreover there was a significant difference for PGE_2 _synthesis between the large and the small particles (#P < 0.01). However, inhibition by indomethacin abolished these significant differences of PGE_2 _production for both types of particles due to the inhibition of PGE_2 _synthesis. These data reveal that the suppressive effect of the small particles on IL-6 release (Figure 3A) was caused by an enhanced PGE_2 _synthesis (Figure 3B) compared to the large particles.

### Bronchoalveolar lavage fluid (BALF) analysis of lung inflammation and injury

**Figure 4:**
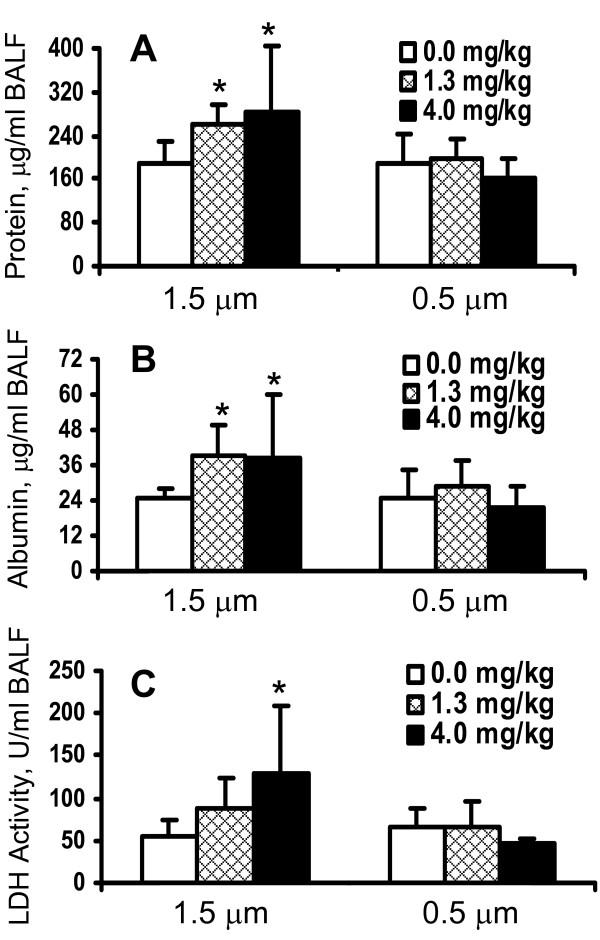
**Injury markers in BALF of rat lungs after instillation of 1.5 and 0.5 μm Fe_2_O_3 _particles (*in vivo *study)**. Healthy WKY rats were intratracheally instilled with 1.5 μm or 0.5 μm Fe_2_O_3 _particles, respectively, at a dose of 1.3 mg/kg body weight and 4.0 mg/kg body weight. At 24 h after instillation pulmonary injury was assessed in the BALF of control and exposed rats. Protein (A) and albumin levels (B) in BALF were determined as markers for vascular permeability, and LDH activity (C) as marker for cytotoxicity. The data are given as mean ± SD with (n = 6) representing 6 animals for each dose and type of particles. * P-value (> 0.05) for significant difference between saline control and Fe_2_O_3 _particle exposed rats.

Because particle number concentration and relative surface area for 0.5 μm Fe_2_O_3 _particles are greater than for the 1.5 μm particles per given treatment concentration, one would expect greater phagocytosis of the smaller particles, and thus, greater injury and inflammation *in vivo*. In order to evaluate relative *in vivo *toxicity and inflammatory potential of 0.5 μm versus 1.5 μm particles, healthy WKY rats were intratracheally instilled with these Fe_2_O_3 _particles, respectively, at a dose of 1.3 mg/kg body weight and 4.0 mg/kg body weight. At 24 h after instillation pulmonary injury was assessed by analysis of injury markers in the BALF of control and exposed rats. As shown in Figure 4A and contrary to what was expected, the content of protein in the BALF increased significantly in the rats exposed to 1.5 μm particles at both dose-levels, whereas that in BALF of rats exposed to 0.5 μm did not change. Similarly, the content of albumin in the BALF was also elevated following exposure to the 1.5 μm particles at both dose-levels, but not after exposure to the 0.5 μm particles (Figure 4B), confirming the larger particles' effect on the protein content of BALF. Moreover, the LDH activity as a marker for cell integrity slightly increased only in rats exposed to 1.5 μm Fe_2_O_3 _particles at 4.0 mg dose-level (Figure 4C).

**Figure 5:**
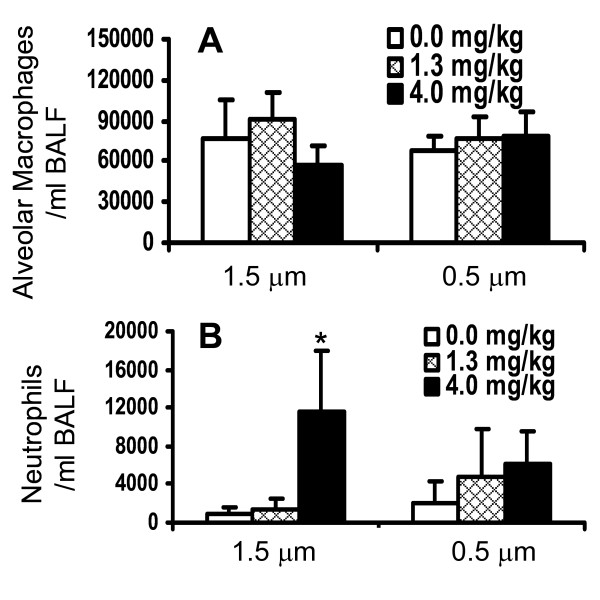
**Inflammatory cells in BALF of rat lungs after instillation of 1.5 and 0.5 μm Fe_2_O_3 _particles (*in vivo *study)**. Healthy WKY rats were intratracheally instilled with 1.5 μm or 0.5 μm Fe_2_O_3 _particles, respectively, at a dose of 1.3 mg/kg body weight and 4.0 mg/kg body weight. At 24 h after instillation pulmonary immune cells such as alveolar macrophages (A) and neutrophils (B) were assessed in the BALF of control and exposed rats. The data are given as mean ± SD with (n = 6) representing 6 animals for each dose and type of particles. * P-value (< 0.05) for significant difference between saline control and Fe_2_O_3 _particle exposed rats.

No change in lavageable AM was noted with either particle (Figure 5A). The number of neutrophils in BALF increased significantly after exposure of the rats to the 1.5 μm particles at a dose of 4 mg/kg body weight, whereas the 0.5 μm particles did not induce an influx of neutrophils (Figure 5B). The instilled particles were readily taken up by AM during the exposure regardless of the size difference. This was apparent when examining BALF cell differential slides under light microscopy (not shown). These data indicate that the large 1.5 μm but not small particles induced an inflammatory response and increased vascular permeability in the rat lungs at the highest dose tested.

## Discussion

There is some evidence to believe that soluble PM-associated iron at low concentrations may have inhibitory effect on PM-induced inflammation [13], however, the mechanism and role of soluble iron or other metals in modulating solid particulate core-induced inflammation are unknown. Because iron is ubiquitously present in ambient PM with differing water solubility [13,14] its role in modulating PM-induced inflammatory responses becomes critical in the toxicity of ambient PM. The present study was designed to determine the role of leachable iron in particle core-induced inflammatory responses *in vitro *within AM and *in vivo *in rats using Fe_2_O_3 _particles of two different sizes and dissolution kinetics. The particles we used were spherical with a porous structure and a rough surface area as outlined in Figure 2. We report here that while Fe_2_O_3 _particles are insoluble extracellularly, intracellular dissolution do occur with the IPD being greater for 0.5 than 1.5 μm Fe_2_O_3 _particles, as expected based on the difference in the specific surface area (Table 1 and Figure 1). For the biologic response of the cells we have studied the effect of the particles on the release of IL-6 as inflammatory marker and on the intracellular synthesis of PGE_2 _as immune-modulating and anti-inflammatory mediator also involved in resolution of inflammation. We have focused specifically on responses of IL-6 because of its importance as acute phase protein as well as pro- and anti-inflammatory cytokine. With regard to these properties, a close relationship between IL-6 and PGE_2 _via an IL-10 dependent mechanism has been recently reported [30]. While only the large 1.5 μm but not the small 0.5 μm Fe_2_O_3 _particles caused a substantial IL-6 release from AM (Figure 3A), cellular PGE_2 _production was much greater with the small 0.5 μm Fe_2_O_3 _particles compared to the large ones (Figure 3B). This difference is emphasized when the relative effects of large and small particles for IL-6 release and PGE_2 _synthesis are opposed (Figure 3). Inhibition of particle-induced PGE_2 _by COX inhibitor indomethacin further enhanced the production of the pro-inflammatory cytokine IL-6 by both sizes to similar levels, so that the significant difference between both particles types was not anymore observed (Figure 3A). Due to this observation we suggest that soluble iron arising from dissolved iron particles within AM modulates particle-induced IL-6 production via PGE_2_. Further, *in vivo *intratracheal instillation of 1.5 μm particles with a lower IPD rate induced lung inflammation and injury in rats while 0.5 μm particles were less effective, consistent with production of IL-6 *in vitro*. These *in vitro *and *in vivo *data together demonstrate that dissolved intracellular iron modulates pulmonary inflammatory responses induced by insoluble Fe_2_O_3 _particle core in rats.

To examine the role of dissolved iron, we chose physicochemical uniform Fe_2_O_3 _particles with two different sizes and, hence, two different specific surface areas such that when incubated at equal mass concentration with AM, smaller 0.5 μm particles will cause greater release of soluble iron than 1.5 μm particles. Our AM culture data confirm that smaller Fe_2_O_3 _particles dissolve more iron intracellularly than particles of 1.5 μm size owing to the differences in the specific surface area. Because both sizes of particles are likely to be readily taken up by AM, we could determine the role of intracellular iron-solubility in eliciting particle-induced inflammatory responses *in vitro *and *in vivo*. Since Fe_2_O_3 _particles were minimally soluble extracellularly, the greater dissolution of Fe_2_O_3 _particles within phagolysosomes supports the greater solubility of PM-associated metals in acidic milieu.

PM-associated metals can be released within the alveolar lining fluid or in cells depending upon their level of solubility and thus, within each compartment they can affect unique cell processes. Extracellular metal can also be internalized by cells, or metal can be released from particles within cells after particles have been phagocytosed. In the present study, there was no dissolved iron extracellularly since the particles leached minimally in the medium without cells. However, particles dissolved in a time-dependent fashion only in the acidic milieu of the phagolysosomes of AM. Furthermore, the intracellular dissolved iron was primarily retained in the cells as observed with AM culture data. Since extracellular iron was minimal, it is unlikely that this iron pool modulated AM responses. Iron being ubiquitously and most abundantly present metal in ambient PM with varying solubility, and as being an essential metal that is tightly regulated physiologically by endogenous cellular processes, the modulation of solid particle-induced inflammation by soluble iron becomes critical in understanding ambient PM-induced pulmonary damage. Antagonistic effect of soluble iron in combustion particles induced inflammation has been suggested [13,31]. Intracellular iron can bind to a variety of iron binding proteins, cell membrane transporters and iron sequestering proteins such as transferrin and ferritin [32]. Depending upon the binding affinity of each of these proteins or specific protein binding sites within the same protein, iron may modulate cell signaling processes induced by phagocytosed particles. The fact that dissolved iron released from particles within phagolysosome remained intracellularly without producing marked cellular injury supports the idea that proteins within cells were able to bind released iron.

Externally added vanadium and zinc have been shown to inhibit phosphatases and induce kinase-specific signaling in cells and subsequent inflammation [33]. Hisakawa et al. [34] observed a depletion of PGE_2 _in human synovial fibroblasts by soluble ferric citrate, which could be reversed by the presence of desferrioxamine. Interestingly, these authors found a comparable increase of PGE_2 _by desferrioxamine in absence of ferric citrate. This suppressive effect of soluble ferric citrate on PGE_2 _in fibroblasts [34] contrasts our findings that more soluble iron enhances PGE_2 _synthesis in AM. Reasons for this discrepancy between the two studies may be due to the amount of soluble iron that existed in extracellular milieu in the Hisakawa study. Existence of large amounts of iron in extracellular fluid may overwhelm protein binding capacity and promote Fenton-like reaction. In case of AM treated with carbonyl iron particles, PGE_2 _production was increased [22] as observed in the present study. This increase in PGE_2 _was correlated with low cytotoxicity of iron particles. In addition, iron oxide particles, larger (2.6 μm) than what was used in our study, induced an acute transient inflammation after instillation in human and rat lungs [28]. These authors found an influx of PMN in the lungs and increased levels of protein, LDH, IL-6 and IL-8 in BAL fluid, whereas PGE_2 _was only marginally elevated. Furthermore, there is evidence for a relationship between IL-6 and PGE_2 _production, since exposure of NO_2 _to rats decreased the levels of TNF- and IL-6 in BAL fluids, whereas PGE_2 _production was increased [35]. PGE_2 _may exert anti-inflammatory effect via its influence on IL-10 production [36]. Since both, IL-6 and PGE_2 _have been shown to be associated with inflammatory responses and anti-inflammatory repair mechanisms, the temporality of induction of each mediator, likely play a crucial role in their functionality.

Our data supports the hypothesis that soluble iron within AM modulates particle-core induced inflammatory responses via increased PGE_2 _synthesis and inhibiting IL-6 release. We have recently shown in *in vitro *studies that ultrafine particles induce PGE_2 _production and this effect is modulated by particle size, specific surface area and composition [8,9]. The particle-induced release of PGE_2 _by AM affects neutrophil respiratory burst activity. The supernatants of particle-treated AM, containing PGE_2_, reduced the respiratory burst activity of stimulated neutrophils, which was restored to control level when PGE_2 _production was prevented in AM by COX inhibitors [8]. Likewise, in the present study, the lack of IL-6 release was associated with higher PGE_2 _production by AM incubated with smaller 0.5 μm particles, while larger 1.5 μm particles produced significantly more IL-6 with less remarkable PGE_2_. Since the intracellular dissolution rate of 0.5 μm particles was greater than that of 1.5 μm particles, it can be concluded that the inflammatory response of small Fe_2_O_3 _particles was inhibited due to higher PGE_2 _production caused by higher levels of soluble iron within AM. The role of PGE_2 _in production of IL-6 by AM is further confirmed by restoration of IL-6 release in AM with 0.5 μm Fe_2_O_3 _particles in the presence of PGE_2 _synthesis inhibitor, indomethacin. PGE_2 _has been recently shown to enhance the production of endogenous IL-10 [30] which plays a central role in the down-regulation of pro-inflammatory cytokines such as IL-6 and TNF in dendritic cells [36,37]. We therefore propose that small sized iron oxide particles activate a mechanism which attenuates inflammatory responses to these particles based on an IL-10 dependent cross-regulation between PGE_2 _and IL-6 acting as a protective pathway in phagocytes, which should be analyzed in further studies.

The conclusion that the presence of intracellular soluble iron suppresses PM-induced inflammation is further supported by the *in vivo *assessment of pulmonary inflammatory response in rats following intratracheal instillations of large 1.5 and small 0.5 μm Fe_2_O_3 _particles. One would expect that greater specific surface area and number concentration at a given mass of 0.5 μm Fe_2_O_3 _particles than that of 1.5 μm particles would lead to higher phagocytosis by AM resulting in greater particle-core induced injury and inflammation. However, we observed that neutrophilic inflammation and lung injury were more pronounced in rats instilled with the large Fe_2_O_3 _particles than the small particles with the latter resulting in higher release of soluble iron within cells. *In vivo*, human exposure to Fe_2_O_3 _particles of >2.5 μm has been shown to cause inflammation following intrabronchial instillation [28]. Although our particles likely not mimic the characteristics of ambient particles, the present study supports the hypothesis that the amount of soluble iron from particulate matter can modulate particle core-induced inflammatory responses.

## Conclusions

In conclusion, increased intracellular soluble iron may modulate Fe_2_O_3 _particle-induced IL-6 release and inflammatory response via PGE_2 _production by AM. This is based on the evidence that greater solubility of 0.5 μm particles resulting in higher intracellular soluble iron was associated with higher PGE_2 _production and suppressed IL-6 release when compared to 1.5 μm particles. The suppressive effect of intracellular released soluble iron on particle-induced inflammation has implication on how ambient PM-associated but soluble metals influence pulmonary toxicity of ambient PM.

## Competing interests

The authors declare that they have no competing interests.

## Authors' contributions

IBS, WGK, KLM, MSB and UPK conceived and designed the experiments; IBS, WGK, ND MCS, PM, MSB and UPK performed experiments; IBS, WGK, UPK performed data analysis; IBS, WGK, KLM and UPK made substantial contributions to writing the manuscript.

## Disclaimer

The research described in this article has been reviewed by the National Health and Environmental Effects Research Laboratory, US Environmental Protection Agency and approved for publication. Approval does not signify that the contents necessarily reflect the views and the policies of the agency nor does mention of trade names or commercial products constitute endorsement or recommendation for use.
